# Evaluation of deep learning-based deliverable VMAT plan generated by prototype software for automated planning for prostate cancer patients

**DOI:** 10.1093/jrr/rrad058

**Published:** 2023-08-22

**Authors:** Noriyuki Kadoya, Yuto Kimura, Ryota Tozuka, Shohei Tanaka, Kazuhiro Arai, Yoshiyuki Katsuta, Hidetoshi Shimizu, Yuto Sugai, Takaya Yamamoto, Rei Umezawa, Keiichi Jingu

**Affiliations:** Department of Radiation Oncology, Tohoku University Graduate School of Medicine, 1-1 Seiryo-machi, Aoba-ku, Sendai, Miyagi, 980-8574, Japan; Radiation Oncology Center, Ofuna Chuo Hospital, Ofuna 6-2-24, Kamakura, Kanagawa 247-0056, Japan; Department of Radiation Oncology, Tohoku University Graduate School of Medicine, 1-1 Seiryo-machi, Aoba-ku, Sendai, Miyagi, 980-8574, Japan; Department of Radiation Oncology, Tohoku University Graduate School of Medicine, 1-1 Seiryo-machi, Aoba-ku, Sendai, Miyagi, 980-8574, Japan; Department of Radiation Oncology, Tohoku University Graduate School of Medicine, 1-1 Seiryo-machi, Aoba-ku, Sendai, Miyagi, 980-8574, Japan; Department of Radiation Oncology, Tohoku University Graduate School of Medicine, 1-1 Seiryo-machi, Aoba-ku, Sendai, Miyagi, 980-8574, Japan; Department of Radiation Oncology, Aichi Cancer Center Hospital, Kanokoden 1-1, Chikusa-ku, Nagoya, Aichi, 464-8681, Japan; Department of Radiological Technology, Keio University, Shinanomachi 35, Shinjuku-ku, Tokyo 160-8582, Japan; Department of Radiation Oncology, Tohoku University Graduate School of Medicine, 1-1 Seiryo-machi, Aoba-ku, Sendai, Miyagi, 980-8574, Japan; Department of Radiation Oncology, Tohoku University Graduate School of Medicine, 1-1 Seiryo-machi, Aoba-ku, Sendai, Miyagi, 980-8574, Japan; Department of Radiation Oncology, Tohoku University Graduate School of Medicine, 1-1 Seiryo-machi, Aoba-ku, Sendai, Miyagi, 980-8574, Japan

**Keywords:** radiotherapy, deep learning, auto planning, artificial intelligence, prostate cancer

## Abstract

This study aims to evaluate the dosimetric accuracy of a deep learning (DL)-based deliverable volumetric arc radiation therapy (VMAT) plan generated using DL-based automated planning assistant system (AIVOT, prototype version) for patients with prostate cancer. The VMAT data (cliDose) of 68 patients with prostate cancer treated with VMAT treatment (70–74 Gy/28–37 fr) at our hospital were used (*n* = 55 for training and *n* = 13 for testing). First, a HD-U-net-based 3D dose prediction model implemented in AIVOT was customized using the VMAT data. Thus, a predictive VMAT plan (preDose) comprising AIVOT that predicted the 3D doses was generated. Second, deliverable VMAT plans (deliDose) were created using AIVOT, the radiation treatment planning system Eclipse (version 15.6) and its vender-supplied objective functions. Finally, we compared these two estimated DL-based VMAT treatment plans—i.e. preDose and deliDose—with cliDose. The average absolute dose difference of all DVH parameters for the target tissue between cliDose and deliDose across all patients was 1.32 ± 1.35% (range: 0.04–6.21%), while that for all the organs at risks was 2.08 ± 2.79% (range: 0.00–15.4%). The deliDose was superior to the cliDose in all DVH parameters for bladder and rectum. The blinded plan scoring of deliDose and cliDose was 4.54 ± 0.50 and 5.0 ± 0.0, respectively (All plans scored ≥4 points, *P* = 0.03.) This study demonstrated that DL-based deliverable plan for prostate cancer achieved the clinically acceptable level. Thus, the AIVOT software exhibited a potential for automated planning with no intervention for patients with prostate cancer.

## INTRODUCTION

Highly conformal radiation therapies, such as intensity-modulated radiotherapy (IMRT) and volumetric-modulated arc radiotherapy (VMAT), can improve the conformance of the dose distribution to the planning target volume (PTV) and reduce the dose to the organs at risks (OARs) [1, 2]. These technologies provide complex dose distribution with a sharp gradient. These IMRT/VMAT plans require an inverse planning process to search for treatment machine parameters with clinically acceptable dose distributions [3]. Inverse planning has multiple parameter adjustments and dose calculation processes. Owing to its basic property of inverse planning, even experienced planners can spend hours tuning the optimization parameters and calling the optimization algorithms multiple times to create a clinically acceptable plan. In addition, this skill-based planning technique causes variations in plan quality among planners [4].

To tackle this issue, a deep learning (DL)-based three-dimensional (3D) voxel-level dose prediction was aggressively developed toward an artificial intelligence (AI)-based automatic planning system [5–7]. A typical DL dose prediction technique uses a convolutional neural network model that receives a 2D or 3D input in the form of PTV/OAR contours with/without planning computed tomography (CT) and produces a voxel-level predicted dose distribution (preDose) as its output [8–10].

Nguyen *et al*. assessed the feasibility of 2D-U-net-based 3D voxel-level dose prediction for patients with prostate cancer. The prediction error of OARs was <5% [11]. Recently, Gronberg *et al*. proposed a 3D densely connected U-net with dilated convolutions and reported that the prediction error of PTV was within 3% for patients with head and neck cancer [12]. These studies demonstrated that DL-based 3D dose prediction exhibited reasonable prediction accuracy for various clinical sites. However, it remained unclear whether preDose could be created via radiotherapy treatment planning system (TPS) (e.g. deliverable plan or not). To solve this issue, several methods were proposed. Fan *et al*. performed automatic treatment planning using preDose with a simple objective function for all voxels indifferently implemented in an open source cross-platform radiation treatment planning toolkit (matRad) [13]. As they used open source TPS to create the RT plan, it could not be implemented in an actual RT machine [13]. Xia *et al*. proposed using the DVH metrics generated from the preDose as objective functions for automatic RT planning [14]. They used a commercial TPS (Pinnacle) and relied on DVH metrics for inverse planning [14], reporting that optimization may not be fully achieved using preDose. Miki *et al*. proposed automatic RT planning using dose-based structures created from preDose [7]. This method can be applied for inverse planning methods across all commercially available TPS. However, creating the multiple dummy regions of interest (i.e. dose-based structures)—for inverse planning and optimizing objective functions for inverse planning—is expensive.

Recently, a prototype of commercial AI-based auto planning assistant system (AIVOT, AiRato. Inc., Yokohama, Japan) was released. This system can efficiently generate multiple dose structures generated from preDose. Furthermore, it can generate a deliverable plan that can reproduce the preDose using the vender-supply objective functions. Thus, this study aimed to clarify the dosimetric accuracy of a DL-based deliverable plan using AIVOT for patients with prostate cancer.

## MATERIALS AND METHODS

### Patient characteristics

This retrospective study was approved by our institutional review board (2022-1-220) and included 68 patients who received radiotherapy with VMAT in our hospital (training, *n* = 55; test, *n* = 13). All patients were treated in our hospital from 2018 to 2022. The total dose was 70–74 Gy/28–37 fractions at the discretion of the radiation oncologists. Planning CT images were acquired using SOMATOM Definition AS+ (Siemens, Forchheim, Germany) with a matrix size of 512 × 512, slice thickness of 2.0 mm and pixel size of 1.27 mm.

All contours were delineated by radiation oncologists. PTV was created by adding a 6-mm margin in all directions (and 5 mm in the posterior direction) to the clinical target volume (CTV). CTV was delineated to include the prostate with/without the base of the seminal vesicles and divided into two parts: PTV 1 was the volume calculated by excluding the rectum from PTV, while PTV 2 was the overlap volume of PTV and rectum. Moreover, 95% of PTV1 received the prescribed dose (i.e. D95@PTV1). Rectum located at the level of the PTV and 6 mm outside of the PTV on the CT images was contoured. The bladder was entirely contoured. The beam arrangement of one full arc VMAT was the same in all the patients.

### Treatment planning

For each patient, VMAT plan was created using one full arc beams using Eclipse commercial treatment system version 15.6 (Varian Medical systems, Palo Alto, USA). All plans were calculated using an Acuros XB algorithm. The dose grid size was 2 mm. The final contours and treatment planning were carefully reviewed and approved by our radiotherapy team, comprising experienced radiation oncologists and physicists. The dose constraints for each plan are summarized in Table 1. The clinically approved VMAT plans (cliDose) were used as ‘ground truth’ plans for training and testing. It should be noted that training data were checked again by experienced medical physicists, and treatment plans that met the clinical protocol but were not of high quality were replanned by experienced physicists. That is, in practice, some clinical plans, although fit for clinical use, could be finetuned further in certain DVH parameters (such as DVH parameters of the rectum, bladder and PTV) to improve to the maximum limit possible (the limits of such finetuning are not fully known, rendering it impossible to predict them in advance). Sasaki *et al*. showed that the plan quality may be improved by the commercial software that presents the limit point [15]. In addition, the clinical plans whose normalization was different from the clinical protocol were modified to match the clinical protocol without majorly changing the plan quality (from D95@PTV to D95@PTV1). The dosimetric data in the training and test clinical plans are shown in Table 2.

**Table 1:** Dose constraints

Target	DVH metric	Constraint
CTV	D98%	>100%
PTV 1	D98%	>90%
	Dmax	<110%
PTV 2	D98%	>90%
	D2%	<100%
Rectum	V70Gy	<10%
	V60Gy	<25%
	V50Gy	<35%
Bladder	V70Gy	<10%
	V60Gy	<25%
	V50Gy	<35%
Right femoral head	V40Gy	<5%
Left femoral head	V40Gy	<5%

PTV1 = PTV without rectum, PTV2 = overlap volume between PTV and rectum.

**Table 2:** Dosimetric data for training and test data

		Training data	Test data	*P*-value
Target	DVH metric	Mean ± SD (%)	Mean ± SD (%)	
CTV	D98%	100.7 ± 0.56	101.2 ± 0.3	0.003
PTV 1	D98%	99.0 ± 0.59	98.8 ± 0.2	0.043
	Dmax	108.1 ± 1.37	107.6 ± 1.0	0.698
PTV 2	D98%	91.6 ± 1.82	93.9 ± 2.7	0.040
	D2%	99.6 ± 1.21	100.0 ± 1.4	1.000
Rectum	V70Gy	2.9 ± 3.25	10.3 ± 4.7	< 0.001
	V60Gy	14.7 ± 4.66	19.7 ± 7.3	0.033
	V50Gy	21.4 ± 5.25	28.6 ± 10.0	0.027
Bladder	V70Gy	10.7 ± 7.44	11.0 ± 6.2	0.168
	V60Gy	16.9 ± 9.62	16.2 ± 8.5	0.588
	V50Gy	22.8 ± 11.89	21.8 ± 11.3	0.839
Right femoral head	V40Gy	0.1 ± 0.68	0.0 ± 0.0	1.000
	D2%	35.8 ± 7.41	32.7 ± 5.4	0.147
Left femoral head	V40Gy	0.0 ± 0.23	0.0 ± 0.1	0.500
	D2%	36.7 ± 6.66	35.3 ± 6.5	0.376

PTV1 = PTV without rectum, PTV2 = overlap volume between PTV and rectum.

### Creation of preDose

**Fig. 1:**
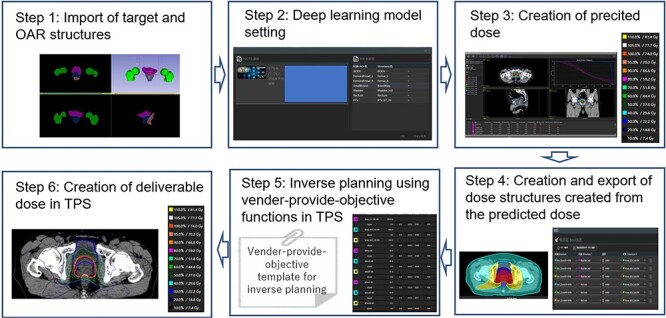
DL-based automated planning workflow.

The overview of auto planning workflow using AIVOT is shown in Fig. 1. There were six processes: step 1 was importing planning CT and the target/OAR structures from TPS; step 2 was setting a DL model connected to the import and preset structure’s name; step 3 was creating preDose via DL; step 4 was creating preDose-based structures and exporting these dose-based structures to TPS; step 5 was inverse planning (optimization) using vender-supply objective functions; and step 6 was obtaining deliDose following the optimization. In step 1, planning CT and the target/OAR structures were imported in a DICOM format. In step 2, it was necessary to associate the contour’s name preset in AIVOT with the corresponding contour’s name in the input structure set. The PTV, rectum, bladder, small bowel, right femoral head, left femoral head and body can be used for DL model setting. Subsequently, a prescription dose was set in the DL model (i.e. D95 of PTV1). The DL model architecture of AIVOT was based on the Hierarchically Densely Connected U-net (HD U-net) proposed by Nguyen *et al*. [6]. Overall, 55 cases were used in the learning process. After completing the learning process, the testing process used the 13 cases that were held out. Additional details are held confidential by the vender and not accessible. After setting the DL model, DL was performed to obtain the preDose (step 3).

### Creation of preDose-based structures for optimization process

**Fig. 2:**
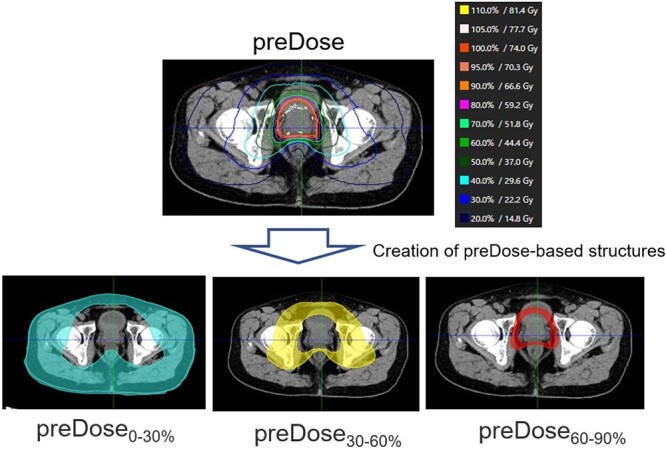
Representative preDose-based structures.

Before creating the deliverable dose (deliDose) based on the predicted dose distribution in TPS, preDose-based structures were required for optimization (i.e. inverse planning) based on the preDose (step 4). Nine preDose-based structures were created using a preinstalled template and the vender-recommended procedure; regions surrounded by 0–30%, 30–60% and 60–90% of the prescribed dose in the body were extracted as preDose_0–30%_, preDose_30–60%_ and preDose_60–90%_, respectively (Fig. 2). In a similar manner, the regions surrounded by 0–30%, 30–60% and 60–90% of the prescribed dose within only the rectum (bladder) were extracted as preDose_Rectum0–30% (preDose_Bladder0–30%), preDose_Rectum30–60% (preDose_Bladder30–60%) and preDose_Rectum60–90% (preDose_Bladder60–90%), respectively. Then, these dose-based structures were exported to TPS.

### Inverse planning using preDose-based structures with vender-supply objective functions

Inverse planning was performed using dose-based structures created from preDose exported from AIVOT and vender-supply objective functions of the target/OARs. At this time, optimization was also performed once. The beam settings were the same as in the training/validation data (i.e. 1 full arc VMAT). The vender-provided objective functions are shown in Table 3. In addition, normal tissue objective function (priority: 80; distance from the target border: 0 cm; start dose: 100%; end dose: 85%; fall-off: 1) was used. DeliDose was created using the same objective functions in all the patients.

**Table 3:** List of objective functions for optimization

Target	Type	Volume (%)	Dose (%)	Priority
CTV	Upper	0.0	100.1	70
	Lower	99.9	102.0	95
PTV 1	Upper	0.0	98.0	95
	Lower	100	93.0	80
	Mean	–	94.3	50
PTV 2	Lower	100	102.0	95
PTV-CTV	Upper	0.0	100.1	70
preDose_0–30%_	Upper	0.1	25.0	80
preDose_30–60%_	Upper	0.0	58.0	70
preDose_60–90%_	Upper	0.0	90.0	50
preDose_103–108%_	Upper	0.0	90.0	50
preDose_bladder_0–30%_	Upper	0.0	30.0	60
preDose_bladder_30–60%_	Upper	0.1	60.0	55
preDose_bladder_60–90%_	Upper	0.0	90.0	50
preDose_rectum_0–30%_	Upper	0.0	25.0	70
preDose_rectum_30–60%_	Upper	0.0	55.0	60
preDose_rectum_60–90%_	Upper	0.0	90.0	50

PTV1 = PTV without rectum, PTV2 = overlap volume between PTV and rectum.

### Evaluation

The DVH parameters used for dose constraints in clinical practice (Table 1) were calculated for preDose and deliDose. For the quantitative evaluation of dose distribution, we calculated the isodose volume dice similarity coefficient (DSC) of the dose distribution (dose interval: 20%) and conformity index (CI) and gradient index (GI) [16, 17]. CI is defined as:


(1) \begin{equation*} \mathrm{CI}=\frac{{{\mathrm{TV}}^2}_{\mathrm{RI}}}{\left(\mathrm{TV}\times{V}_{\mathrm{RI}}\right)} \end{equation*}

where TV is the target volume, TV_RI_ is the target volume covered by the prescription isodose and *V*_RI_ is the total volume covered by the prescription isodose. GI is defined as the ratio of volume of 50% isodose line and volume of prescription isodose line. A blinded scoring of the deliDose and cliDose plans was performed by an expert radiation oncologist. Plan rating performed using an assessment form included a 5-point clinically usable level scale [poor (1)–excellent (5), 3 = minimum]. In addition, using a 3D diode array detector (ArcCHECK, SunNuclear, Melbourne, FL, USA), we assessed patient-specific QA by evaluating the global gamma passing rate (%/mm) for the absolute dose at the set criteria of 3%/2 mm. The dose threshold was 10%. To ensure the positional accuracy of the setup, all measurements were irradiated with a 10 × 10 cm^2^ field defined by collimator jaws. We assessed the VMAT plan complexity using modulation complexity scores (MCSv) to evaluate the multileaf collimator movement during the delivery of VMAT plans [18, 19]. The statistical differences among them were determined via Wilcoxon signed-rank test using JMP version 16.0.0 (SAS Institute, Cary, USA). Differences with a *P*-value of <0.05 were considered significant.

## RESULTS

The DVH parameters for preDose, deliDose and cliDose in all the patients are summarized in Table 4. In addition, the mean and absolute difference in the DVH parameters between preDose or deliDose and cliDose is shown in Table 5. The dose difference means to subtract the DVH parameter in preDose (or deliDose) from that in cliDose. The absolute dose difference is defined as the absolute value of the difference. The average absolute dose difference of all DVH parameters for the target tissue (CTV and PTV) between cliDose and preDose across all patients was 1.98 ± 2.22% (range: 0.04–9.36%), while that for the OARs was 2.04 ± 2.48% (range: 0.00–14.87%).

**Table 4:** Summary of DVH parameters for preDose, deliDose and cliDose

Target	DVH metric	preDose (%)	deliDose (%)	cliDose (%)	*P*-value (preDose vs cliDose)	*P*-value (preDose vs deliDose)	*P*-value (deliDose vs cliDose)
		Mean ± SD (%)	Mean ± SD (%)	Mean ± SD (%)			
CTV	D98%	101.1 ± 1.15	101.8 ± 0.89	101.2 ± 0.35	0.013	0.588	0.052
PTV 1	D98%	98.2 ± 1.84	98.2 ± 0.36	98.9 ± 0.18	0.027	0.014	< 0.001
	Dmax	104.7 ± 0.92	109.1 ± 1.07	107.6 ± 0.92	< 0.001	< 0.001	0.001
PTV 2	D98%	89.3 ± 2.12	90.6 ± 0.90	93.4 ± 2.16	0.012	0.005	0.004
	D2%	99.2 ± 0.56	100.4 ± 0.40	99.6 ± 0.39	< 0.001	0.196	0.001
Rectum	V70Gy	8.7 ± 3.85	8.4 ± 3.37	10.0 ± 4.15	0.735	0.147	0.027
	V60Gy	18.3 ± 6.34	16.6 ± 5.85	19.8 ± 6.50	< 0.001	0.191	0.002
	V50Gy	25.0 ± 6.98	23.9 ± 6.73	28.9 ± 9.26	0.017	0.048	0.027
Bladder	V70Gy	12.1 ± 6.17	10.7 ± 5.95	11.3 ± 6.06	< 0.001	0.040	0.027
	V60Gy	16.8 ± 7.70	15.2 ± 7.74	16.1 ± 8.45	< 0.001	0.017	0.001
	V50Gy	22.2 ± 9.66	20.2 ± 9.72	21.7 ± 11.3	< 0.001	0.040	0.001
Right femoral head	V40Gy	0.0 ± 0.00	0.0 ± 0.00	0.0 ± 0.00	1.000	1.000	1.000
	D2%	32.7 ± 4.58	32.8 ± 5.96	32.7 ± 5.39	0.414	0.787	0.946
Left femoral head	V40Gy	0.0 ± 0.00	0.0 ± 0.00	0.0 ± 0.01	1.000	1.000	1.000
	D2%	34.2 ± 5.95	37.5 ± 6.41	35.3 ± 6.54	0.006	0.266	0.068

PTV1 = PTV without rectum, PTV2 = overlap volume between PTV and rectum, preDose = predicted dose distribution, deliDose = deliverable dose distribution, cliDose = clinical dose distribution

**Table 5:** Summary of relative and absolute differences in DVH parameters between preDose or deliDose and cliDose

Target	DVH metric	preDose - cliDose	deliDose - cliDose	|preDose - cliDose|	|deliDose - cliDose|
		Mean ± SD (%)	Mean ± SD (%)	Mean ± SD (%)	Mean ± SD (%)
CTV	D98%	−0.01 ± 1.08	0.43 ± 0.91	1.02 ± 0.77	0.79 ± 0.92
PTV 1	D98%	−0.46 ± 1.55	−0.41 ± 0.47	0.74 ± 1.84	0.61 ± 0.46
	Dmax	−1.99 ± 1.87	1.01 ± 1.04	0.61 ± 1.54	1.51 ± 0.87
PTV 2	D98%	−2.69 ± 3.49	−1.82 ± 2.25	4.63 ± 2.71	2.97 ± 1.90
	D2%	−0.22 ± 0.65	0.53 ± 0.64	0.68 ± 0.47	0.79 ± 0.64
Rectum	V70 Gy	−0.91 ± 2.77	−1.13 ± 1.96	2.86 ± 1.98	2.11 ± 1.73
	V60 Gy	−1.07 ± 2.72	−2.16 ± 2.64	2.83 ± 2.06	3.27 ± 2.51
	V50 Gy	−2.70 ± 5.03	−3.46 ± 5.25	5.43 ± 4.20	5.60 ± 5.15
Bladder	V70 Gy	0.54 ± 1.22	−0.43 ± 1.05	1.35 ± 0.86	0.77 ± 1.15
	V60 Gy	0.49 ± 1.19	−0.59 ± 0.93	1.16 ± 1.03	0.88 ± 1.00
	V50 Gy	0.35 ± 2.24	−1.05 ± 1.86	1.75 ± 2.10	1.55 ± 2.08
Right femoral head	V40 Gy	0.00 ± 0.00	0.00 ± 0.00	0.00 ± 0.00	0.00 ± 0.00
	D2%	0.02 ± 2.54	0.13 ± 3.42	2.26 ± 1.67	3.18 ± 2.07
Left femoral head	V40 Gy	0.00 ± 0.01	0.00 ± 0.01	0.00 ± 0.01	0.00 ± 0.01
	D2%	−0.80 ± 3.24	1.49 ± 3.31	2.63 ± 2.65	3.10 ± 2.61

PTV1 = PTV without rectum, PTV2 = overlap volume between PTV and rectum, preDose = predicted dose distribution, deliDose = deliverable dose distribution, cliDose = clinical dose distribution.

The largest difference in absolute doses was 5.43 ± 4.20% in V50 Gy of the rectum (dose difference: −2.70 ± 5.03%). Significant differences were observed in D98% of CTV, D98% of PTV2, V70 Gy of bladder and all the DVH parameters of PTV1 and rectum. Average DSCs for different isodose volume classes 0–20%, 20–40%, 40–60%, 60–80%, 80–100% and 100–120% between preDose and cliDose for all the patients were 0.61 ± 0.06 (range: 0.49–0.71), 0.70 ± 0.05 (range: 0.60–0.76), 0.62 ± 0.06 (range: 0.52–0.71), 0.63 ± 0.05 (range: 0.56–0.73), 0.75 ± 0.08 (range: 0.70–0.82) and 0.95 ± 0.02 (range: 0.93–0.96), respectively.

The average absolute dose difference of all DVH parameters for the target between cliDose and deliDose was 1.32 ± 1.35% (0.04–6.21%), while that for the OARs were 2.08 ± 2.79% (0.00–15.37%). The largest absolute difference was 5.60 ± 5.15% in the V50 Gy of the rectum (dose difference: −3.46 ± 5.25%). Significant differences were observed in all DVH parameters (except for D98% of CTV and all DVH parameters of femoral heads). Average DSCs (range) in each isodose volume for 0–20%, 20–40%, 40–60%, 60–80%, 80–100% and 100–120% between deliDose and cliDose were 0.66 ± 0.06 (0.56–0.74), 0.70 ± 0.05 (0.62–0.79), 0.60 ± 0.08 (0.44–0.76), 0.61 ± 0.08 (0.48–0.76), 0.77 ± 0.04 (0.68–0.82) and 0.95 ± 0.01 (0.94–0.96), respectively. CI for preDose, deliDose and cliDose were 0.83 ± 0.07, 0.82 ± 0.06 and 0.83 ± 0.07, respectively, whereas GI were 3.65 ± 0.14, 3.49 ± 0.08, and 3.64 ± 0.13, respectively. Significant differences were observed between deliDose and cliDose (*P*-value, CI: 0.02, GI: <0.001).

**Fig. 3:**
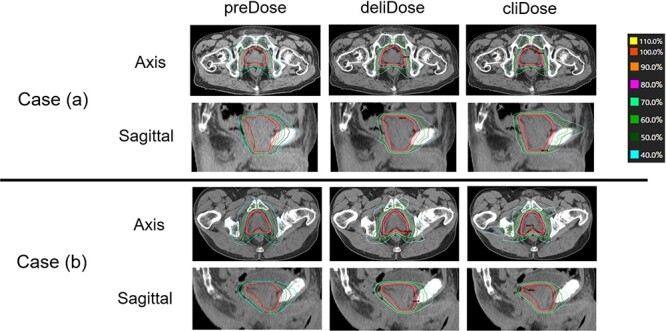
Comparison of preDose, deliDose and cliDose in two cases.

The blinded clinical plan scoring of deliDose and cliDose was 4.54 ± 0.50 and 5.0 ± 0.0, respectively (*P* = 0.03). All plans scored ≥4 points (seven patients: deliDose and cliDose had the same score; six patients: deliDose was 1 point lower than cliDose). Figure 3 shows the preDose, deliDose and cliDose for two cases. Case (a) was a representative case showing the comparable deliDose and cliDose (plan score: deliDose: 5 vs cliDose: 5; average isodose DSC value for each dose interval = 0.80), and case (b) was a representative case in which deliDose is inferior to cliDose (plan score: deliDose: 4 vs cliDose: 5; average isodose DSC = 0.67). The average (range) gamma passing rates for deliDose and cliDose were 96.7 ± 1.7% (94.1–99.1%) and 97.2 ± 1.8% (94.2–99.6%), respectively (*P*-value = 0.44). The average (range) MCSv for deliDose and cliDose were 0.30 ± 0.02 (0.25–0.32) and 0.30 ± 0.06 (0.19–0.36), respectively (*P*-value = 0.59).

## DISCUSSION

We evaluated the dosimetric accuracy of DL-based deliverable plan using AIVOT for patients with prostate cancer. AI-based predicted and deliverable plans were within 6% of dose difference in all the DVH parameters of the target and OAR in all the patients compared with the clinical plan. The AIVOT software exhibited a great potential for automated planning with no intervention for patients with prostate cancer.

The absolute doses value range in preDose and deliDose VMAT plans differed within 6% for all the DVH parameters, target tissue or OARs combinations in all the patients. Regarding the preDose, Nguyen *et al*. evaluated the prediction accuracy of preDose for patients with head and neck cancer using the same DL model (i.e. HD-U-net) as AIVOT. They reported that the prediction error of the OARs was within 6.3% [6]. Our result is consistent with their result (our data: 5.43%). Miki *et al*. evaluated the deliDose prepared in a similar manner in patients with head and neck cancer; the mean dose of the OARs between deliDose and cliDose was approximately within 4–5 Gy. Our result had better prediction accuracy than their result. This may be due to difference in the clinical site (e.g. prostate vs head and neck cancer), consistency of treatment plan quality and DL architecture (e.g. U-net vs HD-U-net).

Regarding the plan quality of deliDose, the deliDose was superior to the cliDose in all DVH parameters for bladder and rectum (Table 4). These results indicated that DL-based automated planning may produce a better plan than a clinically accepted plan in OAR sparing. The reason for this may be due to data cleansing for DL model by replanning the treatment plans that met the clinical protocol but were not of high quality. The blinded physician plan score was >3 across all the treated patients. A physician plan score of >3 is the criteria for clinical use for the deliverable and clinical VMAT plans. However, the average physician plan score for the clinical VMAT plan was significantly higher than that for the deliverable plan (4.54 vs 5.0). In case b (Fig. 3), which is a representative case wherein deliDose is inferior to cliDose, there was no major difference in DVH parameters (within 3%); however, there was a slight difference in GI (deliDose: 3.6 vs cliDose: 3.89). The isodose DSC also showed moderate agreement with the dose ranges <60% (i.e. 0–20%: 0.56; 20–40%: 0.62; 40–60%: 0.53; 60–80%: 0.60; 80–100%: 0.76; and 100–120%: 0.94). The reason that deliverable plan had lower physician plan score may be due to several factors, such as (i) slight increase of hot spot (dose >105%) in PTV (although deliDose had the steeper dose gradient) and (ii) the deviation in dose range (deviations observed <60%). The VMAT plan complexity and reproducibility with linear accelerator were similar for the deliverable and clinical plans; MCSv and the gamma passing rate were similar. Plans created using AI tend to be complicated. Kubo *et al*. evaluated the plan complexity (MCSv) using RapidPlan (RP) (Varian Medical System, a knowledge-based planning system that uses machine learning) [20]. They reported that MCSv for RP was 0.25 ± 0.02 and that for clinical plan was 0.35 ± 0.03, indicating RP may cause more complexity plan owing to increase in smaller segments. This is not consistent with our result. This discrepancy can be due to differences in the optimization process employed by AIVOT and RP. The optimization calculation in AIVOT probably converges more easily because of the preDose-base structure inputs in TPS. Therefore, this simple method may lead to lower complexity than RP.

Taken together, our results indicate that the deliDose VMAT planning module based on AIVOT DL can be introduced for clinical application as a DL-based automated VMAT planning module to treat prostate cancer. To use this system widely, it may be necessary to adapt it to various planning strategies. Despite having nationally accepted practice guidelines, an actual clinical practice is rarely clearly defined in black and white [21]. Kandalan *et al*. evaluated a method for adapting the DL model to different treatment planning practices with minimal input data. To utilize this system in more facilities, it may be necessary to develop a way to adapt a standard AI model built with large-scale data to different strategies in a small amount of data.

The limitations of this study are as follows. First, the feasibility of automatic planning workflow was assessed for one TPS (i.e. Eclipse). Second, the impact of SpaceOAR (Boston Scientific, Marlborough, USA) on AIVOT was not evaluated. Because in our hospital, the SpaceOAR is not administered to patients with prostate cancer who are treated with conventionally fractionated radiotherapy, the patients with SpaceOAR were not evaluated. Third, significant differences were observed between some DVH parameters in the training and test data; probably replanning for cases resulted in improved plan quality. Fourth, RP is already in clinical use as an AI-based optimization calculation system [22, 23]. Currently, RP supports only Eclipse, but AIVOT can, in principle, support any TPS. In the future, we plan to further verify this point by comparing the performance of RP and AIVOT. Fifth, the parameters of the preDose-based structure are set based on vender recommendations. As these settings affect the quality of the deliverable plans, one must evaluate its impact on plan quality in future studies.

## CONCLUSIONS

This study demonstrated that DL-based deliverable plan for prostate cancer achieved the clinically acceptable level. Thus, the AIVOT software exhibited a great potential for automated planning with no intervention for patients with prostate cancer.

## CONFLICTS OF INTEREST

Kadoya had the stock of AiRato. Inc. Jingu has a research grant from AiRato. Inc.

## References

[1] Yamamoto T, Katagiri Y, Tsukita Y et al. Stereotactic radiosurgery for lung cancer with a risk-adapted strategy using the volumetric modulated arc therapy technique: a single arm phase II study. Cancers (Basel) 2022;14:3993. 36010985 10.3390/cancers14163993 PMC9406332

[2] Jingu K, Matsushita H, Yamamoto T et al. Stereotactic radiotherapy for pulmonary oligometastases from colorectal cancer: a systematic review and meta-analysis. Technol Cancer Res Treat 2018;17:153303381879493. 10.1177/1533033818794936 PMC6111389 30145943

[3] Webb S . The physical basis of IMRT and inverse planning. Br J Radiol 2003;76:678–89. 14512327 10.1259/bjr/65676879

[4] Nelms BE, Robinson G, Markham J et al. Variation in external beam treatment plan quality: an inter-institutional study of planners and planning systems. Pract Radiat Oncol 2012;2:296–305. 24674168 10.1016/j.prro.2011.11.012

[5] Kajikawa T, Kadoya N, Ito K et al. A convolutional neural network (CNN) approach for intensity-modulated radiation therapy (IMRT) dose distribution prediction in prostate cancer patients. J Radiat Res 2019;60:685–93. 31322704 10.1093/jrr/rrz051 PMC6805973

[6] Nguyen D, Jia X, Sher D et al. 3D radiotherapy dose prediction on head and neck cancer patients with a hierarchically densely connected U-net deep learning architecture. Phys Med Biol 2019;64:065020. 30703760 10.1088/1361-6560/ab039b

[7] Miki K, Kusters M, Nakashima T et al. Evaluation of optimization workflow using custom-made planning through predicted dose distribution for head and neck tumor treatment. Phys Med 2020;80:167–74. 33189047 10.1016/j.ejmp.2020.10.028

[8] Jhanwar G, Dahiya N, Ghahremani P et al. Domain knowledge driven 3D dose prediction using moment-based loss function. Phys Med Biol 2022;67:185017. 10.1088/1361-6560/ac8d45 PMC9490215 36027876

[9] Yang J, Zhao Y, Zhang F et al. Deep learning architecture with transformer and semantic field alignment for voxel-level dose prediction on brain tumors. Med Phys 2023;50:1149–61. 36434793 10.1002/mp.16122

[10] Kajikawa T, Kadoya N, Tanaka S et al. Dose distribution correction for the influence of magnetic field using a deep convolutional neural network for online MR-guided adaptive radiotherapy. Phys Med 2020;80:186–92. 33189049 10.1016/j.ejmp.2020.11.002

[11] Nguyen D, Long T, Jia X et al. A feasibility study for predicting optimal radiation therapy dose distributions of prostate cancer patients from patient anatomy using deep learning. Sci Rep 2019;9:1076. 30705354 10.1038/s41598-018-37741-x PMC6355802

[12] Gronberg MP, Gay SS, Netherton TJ et al. Technical note: dose prediction for head and neck radiotherapy using a three-dimensional dense dilated U-net architecture. Med Phys 2021;48:5567–73. 34157138 10.1002/mp.14827

[13] Fan J, Wang J, Chen Z et al. Automatic treatment planning based on three-dimensional dose distribution predicted from deep learning technique. Med Phys 2019;46:370–81. 30383300 10.1002/mp.13271

[14] Xia X, Wang J, Li Y et al. An artificial intelligence-based full-process solution for radiotherapy: a proof of concept study on rectal cancer. Front Oncol 2021;10:616721. 33614500 10.3389/fonc.2020.616721 PMC7886996

[15] Sasaki M, Nakaguchi Y, Kamomae T et al. Impact of treatment planning quality assurance software on volumetric-modulated arc therapy plans for prostate cancer patients. Med Dosim 2021;46:e1–6. 10.1016/j.meddos.2021.03.013 33972163

[16] Paddick I . A simple scoring ratio to index the conformity of radiosurgical treatment plans. Technical Note. J Neurosurg 2000;93:219–22. 11143252 10.3171/jns.2000.93.supplement

[17] Paddick I, Lippitz B. A simple dose gradient measurement tool to complement the conformity index. J Neurosurg 2006;105:194–201. 10.3171/sup.2006.105.7.194 18503356

[18] McNiven AL, Sharpe MB, Purdie TG. A new metric for assessing IMRT modulation complexity and plan deliverability. Med Phys 2010;37:505–15. 20229859 10.1118/1.3276775

[19] Masi L, Doro R, Favuzza V et al. Impact of plan parameters on the dosimetric accuracy of volumetric modulated arc therapy. Med Phys 2013;40:071718. 23822422 10.1118/1.4810969

[20] Kubo K, Monzen H, Ishii K et al. Dosimetric comparison of RapidPlan and manually optimized plans in volumetric modulated arc therapy for prostate cancer. Phys Med 2017;44:199–204. 28705507 10.1016/j.ejmp.2017.06.026

[21] Kandalan RN, Nguyen D, Rezaeian NH et al. Dose prediction with deep learning for prostate cancer radiation therapy: model adaptation to different treatment planning practices. Radiother Oncol 2020;153:228–35. 33098927 10.1016/j.radonc.2020.10.027 PMC7908143

[22] Tol JP, Delaney AR, Dahele M et al. Evaluation of a knowledge-based planning solution for head and neck cancer. Int J Radiat Oncol Biol Phys 2015;91:612–20. 25680603 10.1016/j.ijrobp.2014.11.014

[23] Fogliata A, Nicolini G, Clivio A et al. A broad scope knowledge based model for optimization of VMAT in esophageal cancer: validation and assessment of plan quality among different treatment centers. Radiat Oncol 2015;10:220. 26521015 10.1186/s13014-015-0530-5 PMC4628288

